# Autophagy and Mitochondrial Dysfunction in Tenon Fibroblasts from Exfoliation Glaucoma Patients

**DOI:** 10.1371/journal.pone.0157404

**Published:** 2016-07-08

**Authors:** Andrew Want, Stephanie R. Gillespie, Zheng Wang, Ronald Gordon, Carlo Iomini, Robert Ritch, J. Mario Wolosin, Audrey M. Bernstein

**Affiliations:** 1 Department of Ophthalmology, Icahn School of Medicine at Mount Sinai, New York, NY, 10029, United States of America; 2 Department of Pathology, Icahn School of Medicine at Mount Sinai, New York, NY, 10029, United States of America; 3 Department of Pharmacology and Systems Therapeutics, Icahn School of Medicine at Mount Sinai, New York, NY, 10029, United States of America; 4 Einhorn Clinical Research Center, New York Eye and Ear Infirmary of Mount Sinai, New York, NY, 10003, United States of America; University of Iowa, UNITED STATES

## Abstract

**Purpose:**

To test the hypothesis that autophagy dysfunction is involved in exfoliation syndrome (XFS), a systemic disorder of extracellular elastic matrices that causes a distinct form of human glaucoma.

**Methods:**

Fibroblasts derived from tenon tissue discards (TFs) from filtration surgery to relieve intraocular pressure in XFS patients were compared against age-matched TFs derived from surgery in primary open-angle glaucoma (POAG) patients or from strabismus surgery. Differential interference contrast light, and electron microscopy were used to examine structural cell features. Immunocytochemistry was used to visualize LOXL1 and Fibulin-5, lysosomes, endosomes, Golgi, and microtubules. Light scatter, Cyto-ID^TM^ and JC1 flow cytometry were used to measure relative cell size, autophagic flux rate and mitochondrial membrane potential (MMPT), respectively. Enhanced autophagy was induced by serum withdrawal.

**Results:**

In culture, XFS-TFs were 1.38-fold larger (by light scatter ratio, p = 0.05), proliferated 42% slower (p = 0.026), and were morphologically distinct in 2D and 3D culture compared to their POAG counterparts. In extended 3D cultures, XFS-TFs accumulated 8–10 times more Fibulin-5 than the POAG-TFs, and upon serum withdrawal, there were marked deficiencies in relocation of endosomes and lysosomes to the perinuclear area. Correspondingly, the XFS-TFs displayed significant accumulation of the autophagasome marker LC3 II (3.9 fold increase compared to POAG levels, p = 0.0001) and autophagic flux rate as measured by Cyto-ID dye was 53% lower in XFS-TFs than in POAG-TFs (p = 0.01), indicating reduced clearance of autophagasomes. Finally the percent of cells with diminished MMPT was 3–8 times larger in the XFS-TFs than in POAG-TFs (p = 0.02).

**Conclusions:**

Our results provide for the first time a link between XFS pathology to autophagy dysfunction, a major contributor to multiple age related diseases systemically throughout the body, in the brain and in the retina. A diminished capacity for degradation of denatured protein and aging cellular organelles may underpin the development of extracellular protein aggregates in XFS.

## Introduction

Exfoliation syndrome (XFS) is an age-related systemic disorder characterized by the accumulation of amorphous protein aggregates within the extracellular matrix of multiple tissues and organs [1] such as blood vessels, skin, and the gallbladder, kidneys, lungs and heart [2–4]. In the eye, starting from the 50–60 years age interval, the ocular tissues of XFS patients facing the posterior aqueous chamber of the eye, most notably the lens, start accumulating flaky XFS aggregates [5]. Mass Spec Analysis has shown that XFM contains many extracellular matrix proteins, proteases and protease inhibitors including, clusterin, ApoE, latent TGFβ binding proteins, complement activation pathway components, LOXL1, metalloproteases, TIMPs, fibrillins, fibulins and lysosomal hydrolases [6–8].

Through a mechanism not yet understood the proteinaceous aggregates induce the iris epithelium to release pigment granules. Carried along with the aqueous humor flow (the fluid circulation systems that provide nutrients to the internal avascular organs of the eye) the pigment granules and/or extracellular aggregates deposit at the trabecular meshwork, the sieving structure of the eye’s outflow facility. These deposits block fluid outflow, because aqueous humor production is not sensitive to the increased resistance, and a sustained elevated intraocular pressure ensues. As in other sources of pressure-induced glaucoma, the elevated pressure reduces blood supply to the retina leading to retinal nerve cell death and gradual loss of visual fields [9]. XFS is the most common identifiable cause of open-angle glaucoma worldwide [10].

Genetic variants on the lysyl oxidase-like 1 (LOXL1) protein, a matrix cross-linking enzyme that is required for elastic fiber formation, are essential for the development of XFS [11–13]; two single nucleotide polymorphisms (SNPs) in the *LOXL1* gene are associated with 99% of disease. However, not all individuals carrying these SNPs develop XFS; these same variants can be found in a high percentage of the unaffected population. Furthermore, XFS is strongly age-related, being reported almost exclusively in elderly populations, suggesting that cellular changes associated with aging are a critical factor in the ontogeny of XFS pathology [13].

A frequent finding in other age-related diseases involving aggregate accumulations is aberrations in cellular degradation, in particular in the mechanism of autophagy, the mechanism that digests misfolded polypeptides and aging macroscopic cellular components [14–16]. This comparative structural, histochemical and functional study of tenon fibroblasts derived from XFS patients vs. those obtained from POAG patients and young individuals with strabismus demonstrate that in XFS, cells display features associated with dysfunction of autophagy, including one of its most consequential sequels, accumulation of diseased mitochondria [17–19].

## Methods

### Explant outgrowth and cell culture

Following the protocol approved by the Icahn School of Medicine at Mount Sinai Institutional Review Board, 0.02 cm^2^ human tenon capsule samples were obtained for XFS and primary open-angle glaucoma (POAG) patients undergoing trabeculectomy and from young patients undergoing corrective strabismus surgery. All patients and guardians of minors gave informed written consent. Unless indicated otherwise reagent were purchased from Sigma (St, Louis, MO). Tissues were set as explants over a thick Matrigel layer in complete medium, a 1:1 mix of Dulbecco’s modified minimal essential medium and Ham F-12 (DMEM-F12; Invitrogen, Temecula, CA) complemented with 10% fetal bovine serum (FBS; Atlanta Biologicals, Atlanta, GE), ABAM mix and Gentamicin (Invitrogen). Fibroblast outgrew from these explants after 5–10 days. Once outgrowths reached 8–10 mm diameter the cells were harvested with Trypsin (Invitrogen) and serially expanded in complete media for up to 10 passages. At various stages cells were observed and photographed under phase contrast microscopy. The comparative experiments described were obtained with cells in passages 2^nd^ to 8^th^ and experiments were performed with cells at equal passage number. Comparison made in selected features between experiments conducted with cells from the 2^nd^ or 3^rd^ passage and cells from the 8^th^ passage (e.g., light scatter or Fibulin 5 distribution, described below) in the process of accumulating duplicates for statistical analysis, fail to indicate a change in fundamental behavior with cell passage.

In all experiments except for the 3D culture described below, cells were studied following release by Tryple^TM^, washing with PBS, centrifugation and resuspension and plating for 24–48 h in a FBS-free medium, consisting of DMEM-F12 plus RPMI-1640 Vitamin Mix (Gibco), ITS Liquid media supplement (Invitrogen), 1 mg/ml glutathione, 2 mM L- glutamine, 1 mM sodium pyruvate, 0.1 mM non-essential amino acids (Invitrogen) with ABAM and Gentamicin (supplemented-serum free medium or SSFM) on bovine collagen-coated (Purcol; Advanced Biomatrix, Poway, CA) surfaces. Cells were characterized 24 to 48 h later. While serum removal does not constitute a bona fide nutrient starvation, it has been firmly established that induction of autophagy is pronounced with any serum removal protocol [20].

For the generation of 3D culture, TFs from passages 2 to 8 were seeded in 24 well plates on glass coverslips at 10 K cells/cm^2^ and cultured in DMEM/F12 complemented with 1% FBS and 1 mM of the vitamin C precursor 2-0-aD Glucopyranosyl-ascorbic acid (Wako, Osaka, Japan) for one month. Under these conditions fibroblasts accumulate and ‘weave’ their own matrix [21].

All studies described henceforth were performed in a comparative manner with the first 4 lines derived from XFS patients, i.e., without selective exclusion of any XFS cell lines, against the first four cell lines derived from POAG donors. Both sets have a similar age range (Table 1).

**Table 1:** The diagnosis, age, sex and medications of donors.

	Diagnosis	Age	Sex	Medications
1	XFS	56	M	Lumigan, Combigan
2	XFS	68	M	Travatan, Cosopt
3	XFS	81	F	Azopt, Xalatan, Predforte
4	XFS	89	M	P4%, Alphagan, Dorzolomide, Xalatan
5	POAG	56	M	Azopt, Alphagan, Lumigan
6	POAG	62	F	Lumigan, Combigan, Dorzolamide
7	POAG	78	F	Predforte
8	POAG	79	F	Zioptan, Betimol, Simbrinza, Diamox, PI
9	Strabismus	3	M	
10	Strabismus	3	F	
11	Strabismus	4	M	
12	Strabismus	10	F	

### Immunocytochemistry

Cultures seeded on glass coverslips were fixed in 3% PFA for 15 min (2D) or 4% PFA for 30 min (3D), excess PFA was neutralized by incubation with 50 mM ammonium nitrate for 10 min and the coverslips were sequentially subjected to, a) permeabilization with 0.1% Triton X100 for 2 min (2D) or 0.5% Triton X100 for 30 min (3D); b) non-specific site blockade with 3% goat serum for 30 minutes; c) overnight incubation at 4°C with a primary mouse monoclonal (MoAb), a rabbit (RbAb) or a goat (GtAb) polyclonal antibody; d) three 5 min washes with PBS; e) incubation for 1 h with fluorophore–conjugated goat anti-mouse or goat anti-rabbit IgG Abs, as appropriate; e) three 15 min washes; f) DAPI counterstaining; g) mounting on glass slides with Vectashield (Vector lab, Burlingame, CA). Fixed stained samples were observed in an upright Zeiss Axioplan2 microscope or in the super-resolution [22] Leica TCS SP8 STED3X (Buffalo Grove, IL). Live cells were examined in the laser-based Leica SP5 DMI confocal inverted microscope both equipped with high resolution image capture systems.

The primary MoAbs used were, anti-Lamp-1 (clone # 9091; Cell Signaling Technology, CST, Danvers, MA); anti-LOXL1 (sc-166632; Santa Cruz, Dallas, TX); anti-Rab 5 (# 3547), 9 (# 5118), and 11 (# 5589) (CST); and anti-Mannose 6-phosphatase receptor (ab2733; Abcam, Cambridge, MA). Goat anti Fibulin 5 Ab (sc-23062) was from Santa Cruz). Rabbit anti-LC3 was purchased from Abgent (Ap1800A0). Species matched Alexa-488, -647, and cy3-conjugated secondary antibodies were obtained from Molecular Probes (Eugene, Oregon).

### Relative proliferation rate

Cultures in glass bottom dishes at 60–70% confluence were incubated for 4.0 h with 50 μM BrdU, washed with PBS and fixed in 70% ethanol for 10 min. After rehydration in PBS the cultures were reacted sequentially incubated in 1 M HCL for 1 h at 37°C and permeabilized with 0.1% Triton-X100, blocked with 1% BSA and reacted with a 1:200 dilution of an anti-BrdU Ab (CST#5292S) for 2 h at 37°C and after PBS washes, with Alexa 488 conjugate goat anti-mouse IgG for 1 h at RT. After one wash with PBS complemented with 0.5 μg/ml Hoechst 33342, to stain all nuclei and two additional PBS washes, coverslips were mounted on glass slides with Vectashield^TM^. The BrdU and Hoechst fluorescences were imaged in an Olympus IMT2 microscope equipped with a Nikon D90 camera. Images were transferred to Photoshop for automated counting of stained nuclei and labelled indices were calculated as BrdU^+^/ total nuclei using > 400 nuclei per measurement.

### Western blot

XFS and POAG-TFs were seeded in SSFM on collagen. After 48 h cells were collected in RIPA lysis buffer (50 mM Tris HCl, 150 mM NaCl, 1.0% NP-40, 0.5% sodium deoxycholate, 1.0 mM EDTA, 0.1% SDS pH of 7.4) and protease inhibitors, the suspension was sonicated, remaining insoluble material was removed by centrifugation and equal amounts of protein were Western blotted for LC3 and GAPDH.

### TEM

Tenon fibroblasts were seeded on collagen in SSFM in a 100mm dish at 80% confluence. Cells were washed 1X with SSFM and fixed with 2.5% glutaraldehyde (Electron Microscopy Sciences) in 0.1M sodium cacodylate buffer (Electron Microscopy Sciences, Hatfield, PA), pH 7.4 for 2 hours at 4°C. Cells were scraped from the plate with 2 volumes of 3ml fixative each time. Pelleted cells were resuspend in 1.2 ml of fixative and incubated overnight at 4°C. The samples were then post-fixed with 1% osmium tetroxide and dehydrated through a graded series of ethanol. Epon resin (ElectronMicroscopySciences) was used for embedding. Ultrathin sections were cut on an ultramicrotome (Leica Ultracut UCT) and stained with uranyl acetate and lead citrate. The sections were then viewed using a H7650 microscope (Hitachi, Japan).

### Autophagy

Autophagosomes and autolysosomes were spatially examined using a lentivector for expression of a tandem-tagged mCherry-EGFP-LC3BB tracing construct [23] and the puromycin selection gene [24], a generous gift of Dr. Ana Maria Cuervo (Albert Einstein School of Medicine, NYC). Lentiviral particles were produced by co-transfection of HEK293T of the tamden-tagget plasmid with plasmids psPAX2 and pCMV-VSV (Addgene, Cambridge, MA) in the presence of LipoD293 (SignaGen, Ijamsville, MD). Culture supernatants were harvested in two batches in the 24–96 post transfection intervals, the combined supernatants were sieved through 0.45 μm filters and the viral particles were gently pelleted by centrifugation at 30,000 x g for 5 h and resuspended in culture medium. One set of age matched POAG and XFS TF were transduced with this lentivector 24 h after replating in the presence of 6 μg/ml polybrene (Fisher). Stably transduced cells were selected by culture complementation with 2 ug/ml puromycin for 48 h. Cells were cultured in glass bottom plates in SSFM. After 24 h the cells were incubated with 1 μg/ml Hoechst 33342 for 5 min to label nuclei and blue, green and red fluorescence emissions were captured in a in the Leica SP5 DMI confocal microscope equipped with a stage that maintains temperature at 37°C and CO_2_ at 5%.

The Cyto-ID Autophagy Detection Kit (Enzo Life Sciences, Farmington, NY) was used to compare autophagic flux rates between the XFS and POAG derived TF lines. Human TFs were seeded at 10 K cells/cm^2^ in SSFM in 12 well plates and were either treated with 10uM chloroquine for 18 h, at 37°C or left untreated (control). Following incubation, cells were trypsinized, collected by centrifugation, washed and resuspended in Cyto-ID Green stain solution in the dark for 30min at 37°C. After pelleting cells were resuspended in ice cold FACS buffer, consisting of phenol red-free (4-(2-hydroxyethyl)-1-piperazine-ethanesulfonic acid (Hepes)-buffered DMEM/F12 complemented with 5% FBS and 1 μg/ml propidium iodide (PI) and were immediately analyzed in an Accuri6 flow cytometer (BD, Franklin Lakes, NJ). All the flow cytometry measurements excluded any PI^high^ (dead) cells and also all cells whose side light scattering (SSC; a relative measure of cellular granularity) to forward light scattering (FSC; a relative measure of cell size) showed a marked upward deviation from the main ratio (SSC^high^ cells). Such a deviation is a proven indication of apoptosis at early stages, due to membrane crenation [25]. The average fluorescence in each sample was normalized to the average FSC of the sample.

### Mitochondrial membrane potential (MMPT)

TFs were seeded at 20K cells/well in normal medium or in SSFM on collagen-coated 24 well dishes. Four to 48 h later, the medium was complemented with 1 μM JC1 for 90 min at 37°C. Cells were then released by trypsinization, resuspended in FACS medium and live cells with normal SSC/FCS ratio were analyzed by flow cytometry. Emissions were measured at 530 (green) and 585 nm (orange) and green/orange ratios were calculated. Alternatively, cells were seeded in SSFM on glass bottom petri dishes, and stained for 1h with JC1. After washing and a brief exposure to 1 μM propidium iodide (PI) to identify permeable cells, hepes-buffered colorless culture medium was introduced and dual color images were captured in an inverted epifluorescent Olympus IMT2 microscope equipped with a dichroic cube for excitation at 480 nm and continuous emission from 525 to 700 nm and a computer controlled Nikon D90 camera.

### Statistical Analysis

Numerical data are expressed as the mean +/- SD of 3–5 independent experiments each in turn consisting of the average of the four XFS and four POAG cell lines. For three groups (XFS, POAG, and no glaucoma control in the immunostaining of 3D culture) statistical significance was calculated by one-way ANOVA with Bonferroni’s test. For all other figures, statistical significance was calculated by the Student’s t-test.

## Results

### XFS-TFs are larger than POAG-TFs

**Fig 1:**
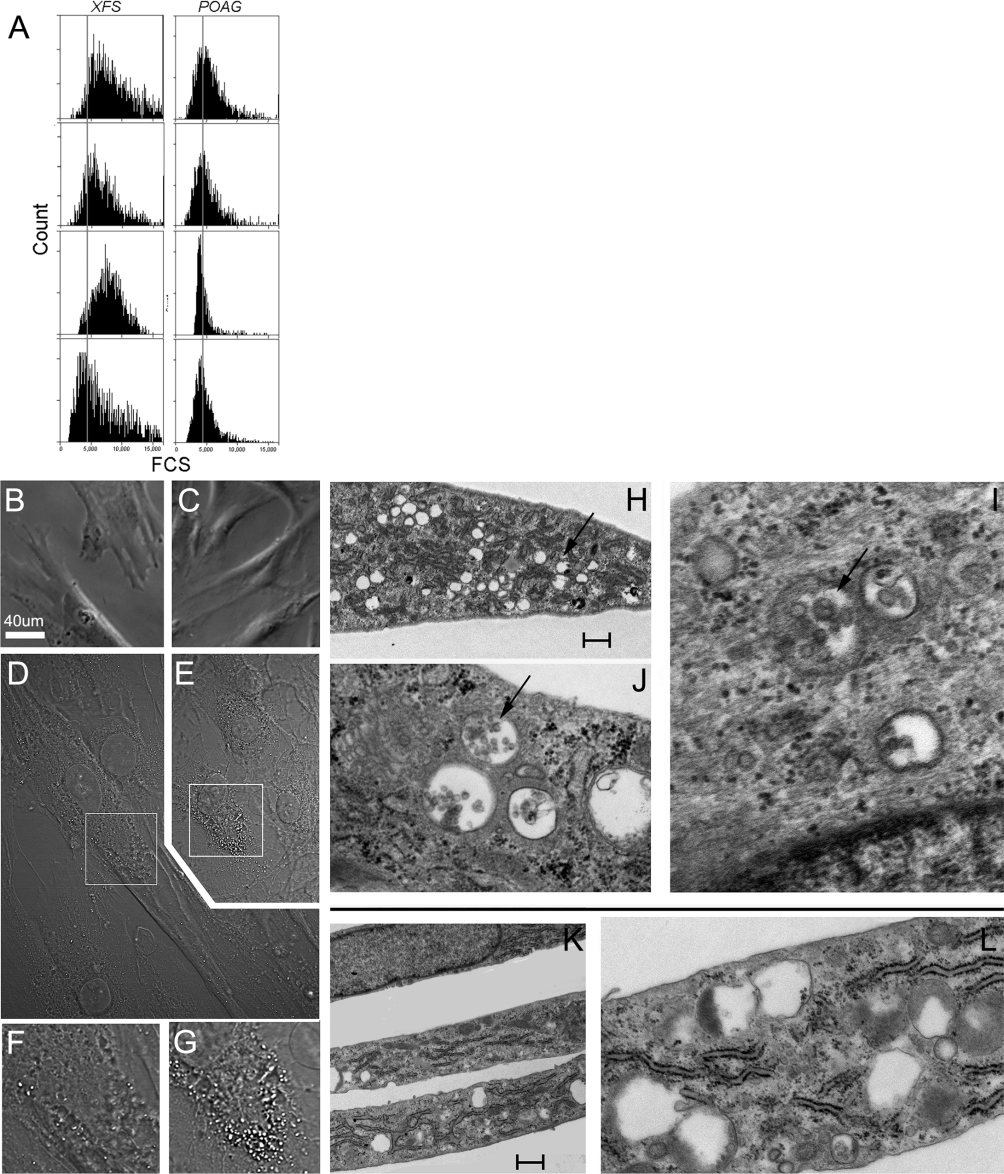
Phenotypic differences between XFS and POAG-TFs. **XFS:** (A) Flow cytometry forward light scatter histograms of XFS-TFs and POAG-TFs. A line traced at an arbitrary value highlights differences between XFS and POAG cells. Note that in the XFS populations most cells have scatter values higher that the line values whereas in the POAG cells scatter values are similarly distributed on both side of the line. B and C) Phase contrast illumination micrographs of XFS-TFs (B) and POAG-TFs (C), bar = 40 μm. D and E) Differential interference contrast illumination micrographs of XFS-TFs (D) and POAG-TFs (E), bar = 40 μm. Magnified XFS-TFs (F) and POAG-TFs (G). Note the difference in the DIC-generated granularity of this perinuclear zone. H-J) Electron micrographs of XFS-TFs K,L) Electron micrographs of POAG-TFs. Note the differences in vacuole density and vacuolar content (arrows in H and K) between both cell types. Bar = 0.5 μm.

The first indication of important phenotypic differences between XFS-TFs and control POAG-TFs was provided by flow cytometry measurements of forward light scattering (FCS), a parameter proportional to cell size, of cells released into suspension (Fig 1A). Comparison of average FCSs between the four XFS and POAG samples where all 8 samples were cultured simultaneously demonstrated that XFS cells are larger than that POAG by 1.38-fold +/- 0.26 (p = 0.05). Additionally, XFS-TFs had a reduced proliferation rate; after 4 hr incubation with BrdU, XFS-TFs displayed 11+/- 3% nuclear labeling (N = 4) compared to 19 +/- 5% (N = 4) of the POAG cells, (p = 0.026). Size and morphological differences were also apparent under phase contrast microscopy (Fig 1B and 1C). XFS-TFs cells appeared larger than the age matched POAG cells serving as controls and did not display the classical spindle shape. This difference persisted across multiple conditions of cell culture and with the increase in generational number to the 10^th^ passage. The results described in this study, nevertheless were obtained with cells in the 2^nd^ to 8^th^ passage.

Closer examination of cellular features of the live cell by laser based differential interference contrast (Nomarski) microscopy suggested also substantial differences in intracellular arrangements between XFS and POAG cells (Fig 1D and 1E). In particular, in the XFS-TFs the interference contrast illumination generated large vacuolar images in the perinuclear proximity quite distinct from the finely defined granularity generated by this analytical method on the POAG-TFs (Fig 1F and 1G). By TEM, the most overt differences we noticed between XFS (Fig 1H, 1I and 1J) and POAG cells (Fig 1K and 1L where a much higher density of vacuoles in the XFS-TFs. Additionally, vacuoles in the XFS cells were mostly replete with cellular debris (arrows), rarely seen in their POAG counterparts.

### XFS-TFs fail to establish the normal fibroblast spatial orientation in 3D cultures and accumulate large amounts of Fibulin-5

**Fig 2:**
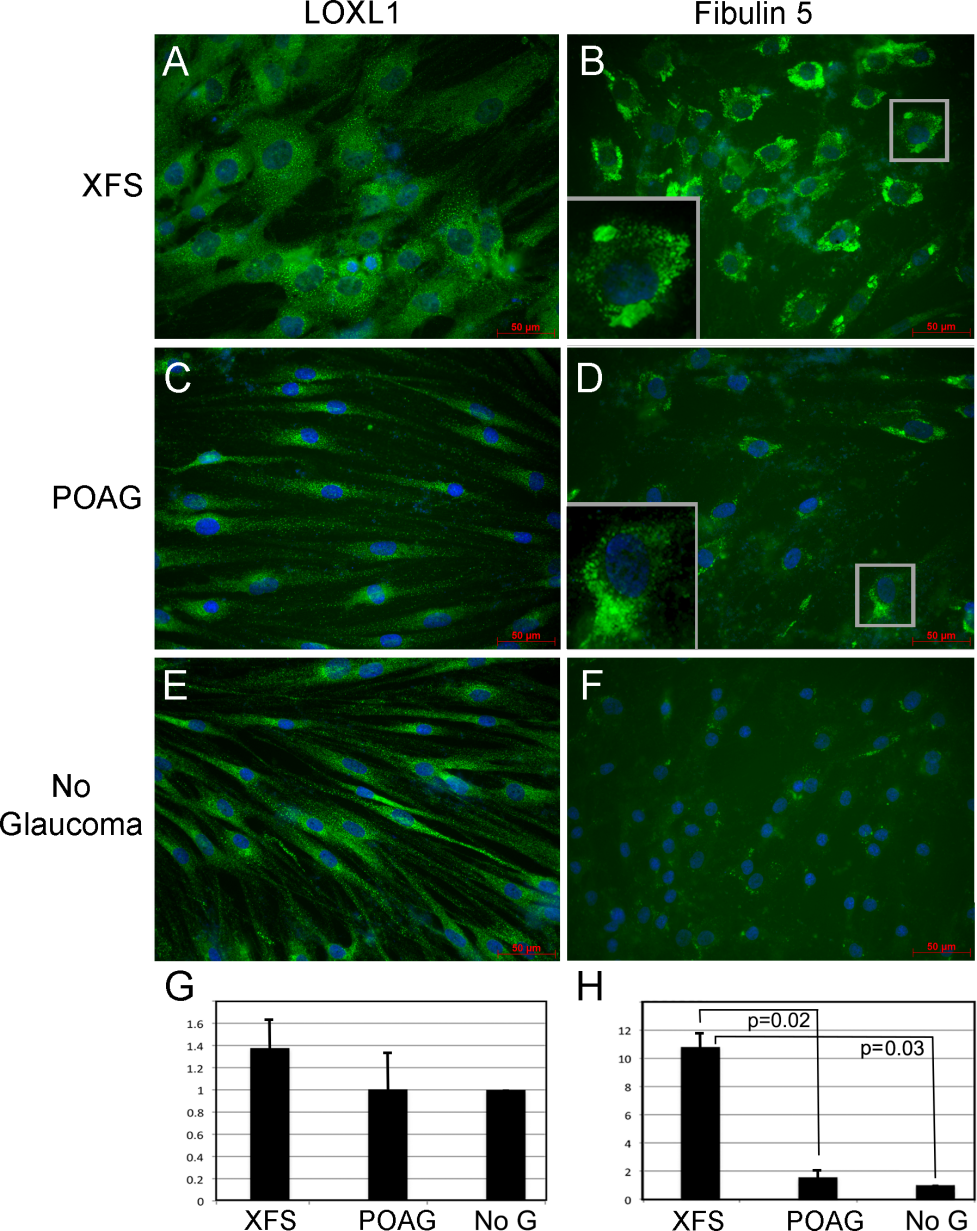
Fibulin-5 and LOXL1 cellular distribution and expression in XFS-TFs in 3D culture. A, C and E) LOXL1 in XFS, POAG and young donors, respectively (LOXL1, green, DAPI, blue). B, D and F) corresponding Fibulin-5 images. Arrows point to potential aggregates. Inset show magnification of LOXL1 and Fibulin-5 cellular distributions. G and H) Images were captured at identical exposure times. Using Metamorph analysis, threshold pixel intensity was set for all images. Area above the threshold was quantified for each image. No glaucoma data was set to 1.0. Statistical significance calculated by one-way ANOVA with Bonferroni’s test. Each patient-derived primary cell line was tested in duplicate for all markers. N = 4 for XFS-TFs, POAG-TFs, and no glaucoma TFs. 10 images from each 3D culture were analyzed.

LOXL1 and Fibulin-5 proteins are essential for the formation of mature, stable elastic fibers [12, 26, 27]. Fibulin-5 simultaneously interacts with LOXL1 and elastin in the LOXL1-mediated cross-linking of elastin fibers formation. To examine the intracellular distribution of these two proteins we resorted to self-forming 3D cultures. In these long term cultures, fibroblasts synthesize and organize self-secreted extra cellular matrix (ECM), hence they provide a robust in vitro model to assess the ability of cells to spatially organize in their surroundings and study the intracellular management of proteins involved in extracellular matrix formation in an environment approaching the *in vivo* conditions [21]. These studies provided intriguing information on these two aspects. After 30 days of 3D culture, as can be directly gleaned from the immunostain micrographs in Fig 2, XFS-TFs displayed a visibly larger surface area than the POAG controls (Fig 2A and 2B compared to Fig 2C–2F) described above, and additionally did not establish the spatial in-parallel alignment that characterizes most fibroblast cultures, exemplified by the cultures of the age-matched POAG controls (Fig 2C and 2D) and the young, no glaucoma controls (Fig 2E and 2F).

The immunostainings revealed distinct cellular distributions of the two elastin fiber associated proteins. LOXL1 was spread in fine puncta throughout the cell in XFS-TFs (Fig 2A) but in controls (Fig 2C and 2E), LOXL1 was centered around the perinuclear area. Fibulin 5 resided in locations more proximal to the cell center (Fig 2B, 2D and 2F). In XFS-TFs, the immunostaining suggested the presence of amorphous aggregates (Fig 2B, arrows, and magnified in insert) compared to controls (Fig 2D and 2F). To quantify the signal using Metamorph analysis, images were captured at identical exposure times. A threshold pixel intensity was set for all images and the area greater than the threshold measurement was quantified. Signal quantification yielded differences for LOXL1 between XFS and POAG or young no glaucoma controls (Fig 2G), which were small and non-statistically significant. In contrast to the invariance of LOXL1, Fibulin-5 expression in the XFS-TFs (Fig 2B, 2D and 2F) was 7.0-fold (p = 0.02) and 10.8-fold +/- 1.0 (p = 0.03) higher than in the POAG or young no glaucoma controls, respectively (Fig 2H). A small difference between the POAG-TFs and young donors was not statistically significant.

### XFS-TF lysosomes fail to relocate to the perinuclear area with autophagy induction

**Fig 3:**
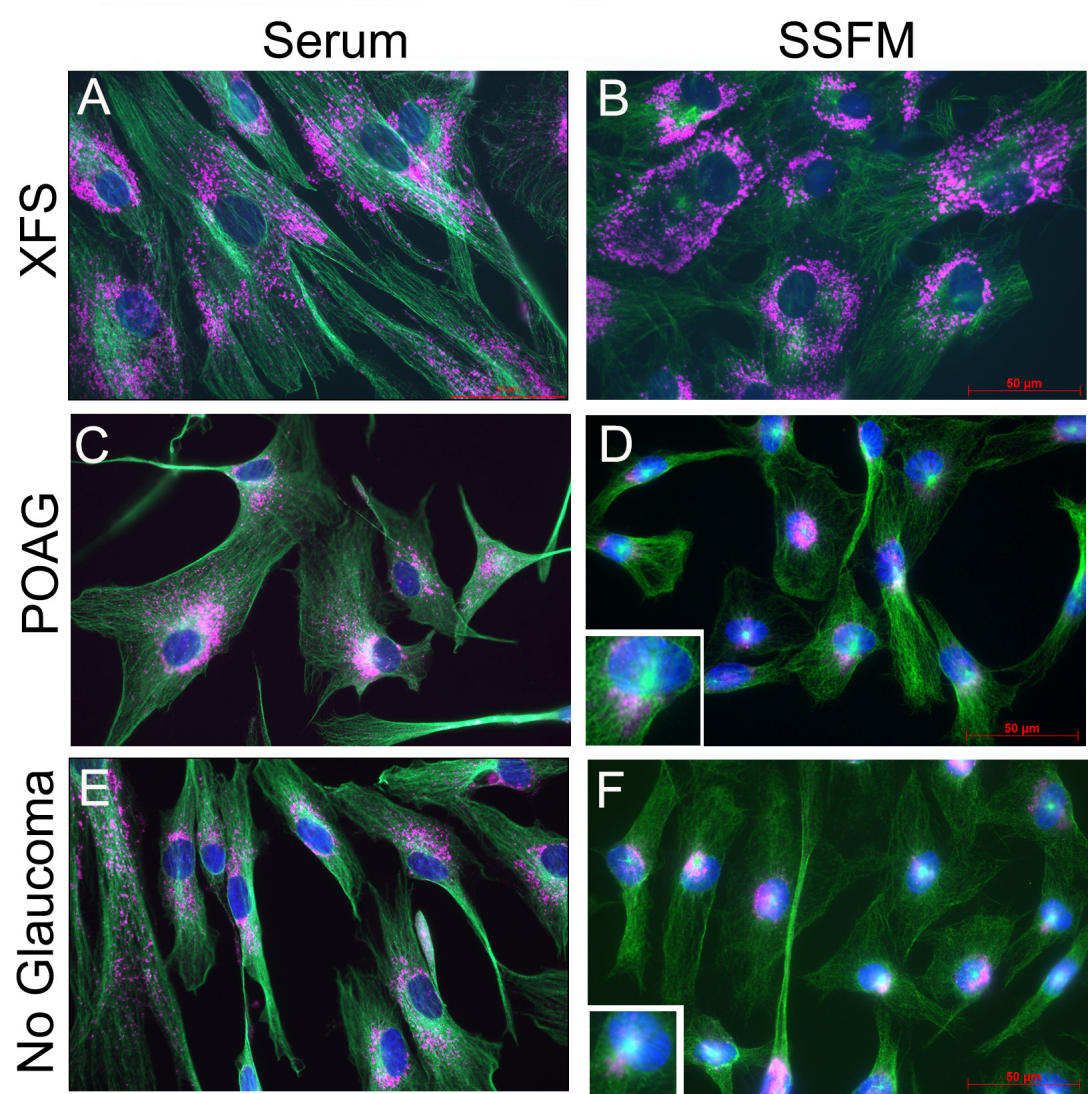
Effect of FBS-withdrawal on spatial distribution of lysosomes. Cells were seeded in serum-containing media (A,C,E) or SSFM on collagen (B,D,F). After 24 hours cells were immunostained for LAMP1 (pink) and β-tubulin (green). A and B) XFS-TFs. C and D) POAG-TFs. E and F) No glaucoma-TFs. Note that in XFS-TFs after serum withdrawal lysosomes fail to relocate to the perinuclear area. Each patient-derived primary cell line was tested in duplicate. N = 4 for each cell type. Bar = 50 μm.

In healthy cells, stress conditions, including serum withdrawal, enhances autophagy. This survival response involves enhanced relocation of lysosomes and autophagosomes to the peri-nuclear area were the latter two can fuse, initially with each other, and ultimately with the concentrating lysosomes. This establishes a transient autolysosome vesicle where misfolded proteins, endocytosed debris and decaying organelles undergo full digestion [28]. Given the large cell size, with an overt increase in vesicular structures, we tested if XFS-TFs demonstrated aberrations in lysosomal/autophagic function. To examine this, XFS-TFs and POAG-TFs were first seeded in either complete medium with 10% serum (Fig 3A, 3C and 3E) or after serum-withdrawal, using the described re-plating protocol described in Methods (Fig 3B, 3D and 3F, starvation medium; SSFM).

To examine the lysosomal compartment we immunostained cells for Lamp1 [29] and, because lysosomes are tethered to microtubules, to provide a spatial frame of reference, cells were double stained for β-tubulin. First, cells were seeded in serum to achieve a baseline level of lysosomal positioning in each cell line. In all three types of cells (XFS, POAG, and young no glaucoma) lysosomes were dispersed when seeded in serum, and most widely dispersed in XFS-TFs (Fig 3A, 3C and 3E). When serum was removed, XFS-TFs lysosomes did not relocate to the peri-nuclear foci, the expected regular cellular response to starvation. In contrast, complete relocation was evident in the age-matched POAG-TFs (compare Fig 3B and 3D, pink stain). Full lysosomal relocation was also seen in the TFs from young donors (Fig 3F).

Lysosomes move along microtubules [30]. Consistent with this knowledge, the perinuclear foci of lysosomal accumulation overlapped with a nodule of intense β-tubulin concentration (inserts in Fig 3D and 3F), which is recognizable as the microtubule organizing center (MTOC;[31]). Likewise, coincident with the failure of lysosomes to migrate to the perinuclear area, an MTOC was substantially less visible or even absent in XFS cells. These correlations suggest that microtubule dysfunction may be at the root of the failure of lysosomes to relocate to the perinuclear zone in XFS-TFs. Since serum-removal elucidated a dramatic lysosomal dysfunction in XFS-TFs, all remaining experiments were performed under these same starvation conditions.

**Fig 4:**
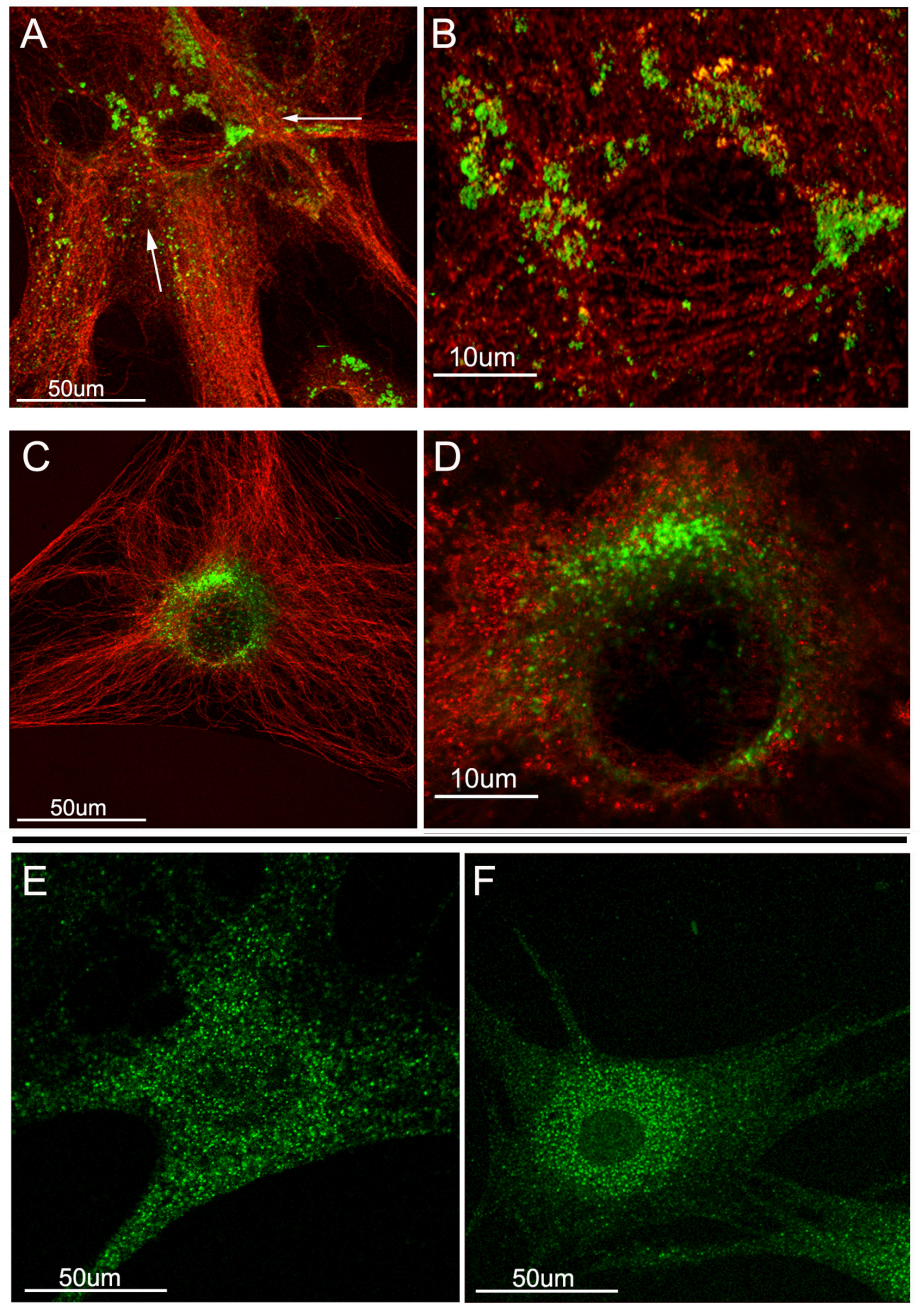
Super resolution micrographs of XFS and POAG-TFs after serum-withdrawal. A-D) LAMP1 (green) and β-tubulin (red). A and B) XFS-TFs. C and D) POAG-TFs, bar = 50 μm or 10 μm. XFS-TFs demonstrate clumped LAMP-1 postive lysosomes compared to POAG-TFs. E and F) LOXL1 immunostained XFS-TFs (E) and POAG-TFs (F) bar = 50um. In XFS-TFs LOXL1 is evenly distributed throughout the cell, compared to in POAG-TFs LOXL1 is centered in the peri-nuclear area. N = 2 for XFS-TFs and POAG-TFs.

To more closely observe the localization of lysosomes in these cells, the novel super-resolution confocal microscopy method [32] was utilized (Fig 4). In agreement with the data in Fig 3, LAMP1 immunostaining in XFS-TFs was delocalized and non-perinuclear (Fig 4A and 4B, green) and occurred as discrete units whereas near the nucleus the stain appeared as large aggregates that could not be resolved even at this high resolution. The stain in the control POAG consisted exclusively of perinuclear distributed fine puncta (Fig 4C and 4D).

Super resolution microscopy was also used to examine LOXL1 localization. In XFS-TFs the punctate dots were evenly distributed throughout the cells (Fig 4E) similar to the distribution described in Fig 2 after long-term culture for 3D generation, whereas in POAG-TFs (Fig 4F) the puncta became highly localized around the nucleus. This latter result suggests again, a deficient intracellular trafficking of secreted proteins in TFs from XFS patients.

### Endocytic pathways are mislocalized in XFS-TFs

**Fig 5:**
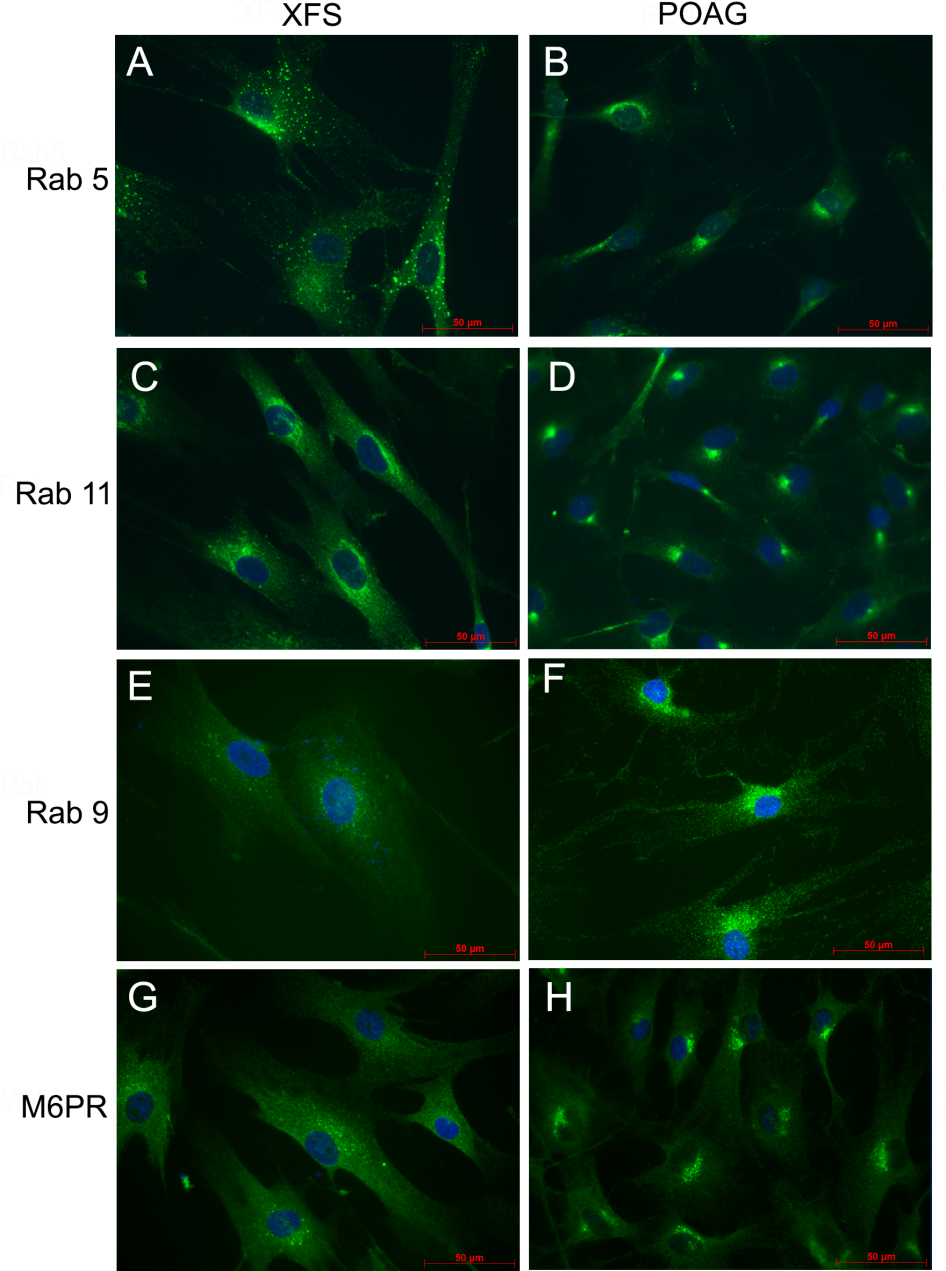
Endosomal marker distribution in XFS-TFs and POAG-TFs. **Left panels.** XFS-TFs. **Right panels.** POAG-TFs. A and B) Rab5, a marker of early endosomes. C and D) Rab11, a marker for recycling endosomes. E-H) Rab9. (E and F) and M6PR (G and H), markers for late endosomes. Bar = 50 μm. Each patient-derived primary cell line was tested in duplicate for all markers. N = 4 for XFS-TFs and POAG-TFs.

To assess if other trafficking organelles were mislocalized in XFS-TFs, cells were fixed and immunostained for proteins that act as reporters of distinct cellular trafficking organelles (Fig 5). In every intracellular trafficking unit examined, XFS-TFs displayed spatial distortions respect to the norm observed in the POAG cells. The stains for Rab 5 (Fig 5A and 5B), an early endosome tracker [33], for rab11 (Fig 5C and 5D), a tracker of recycling endosomes [34], and for rab 9 and the mannose-6-phosphate receptor Fig 5E–5H), markers for late endosome and Golgi, were mostly dispersed throughout the cell, whereas in POAG-TFs, all four stains showed a high concentration at a single peri-nuclear foci. These XFS-TFs images are characteristic of dysfunctional endocytic trafficking pathways observed in cells known to have deficient autophagy and/or specific lysosomal disease [35].

### XFS-TFs demonstrate accumulation of the autophagosomes-bound LC3-II in aggregate form and display reduced autophagic flux

Microtubule-associated protein 1 light chain 3 (LC3) is a critical component for autophagosome development and as its names indicates, for their microtubule dependent migration towards the cell center. The free cytosolic isoform (LC3-I) is converted by lipidation into an isoform (LC3-II), which binds to the developing autophagasomes and helps in the tethering of the maturing autophagosome to microtubules [36]. Both LC3I and LC3II are used as markers for autophagy at different steps that reveal a balance of biogenesis and degradation [37].

**Fig 6:**
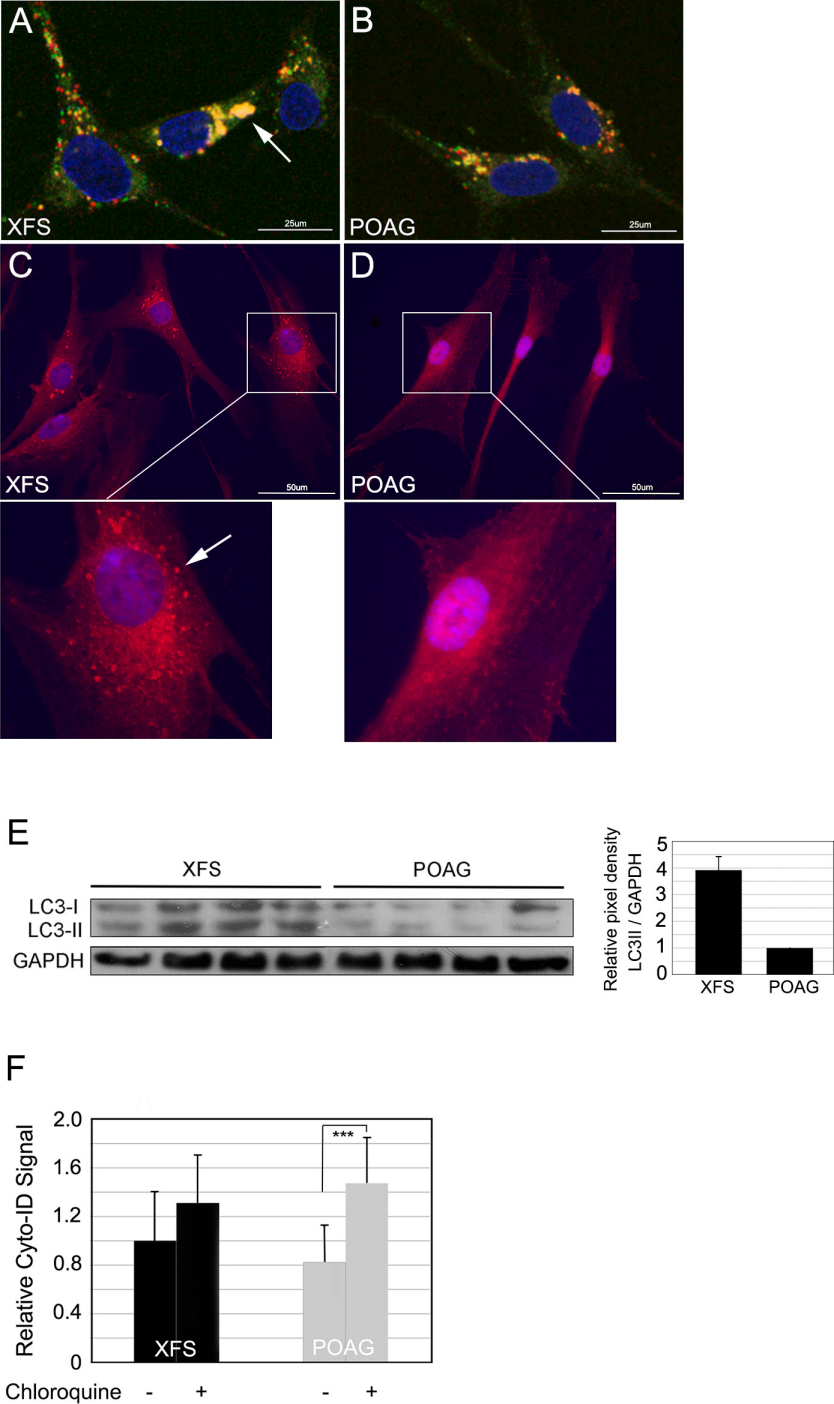
LC3-II and autophagic flux in XFS and POAG-TFs. A and B. Representative images of fluorescence emissions of TFs stably transduced with tandem mCherry-EGFP-LC3B 24 h after induction of starvation. In the XFS cells (A), some of the autophagosomes are located around the nuclei, but a large fraction are present everywhere else in the cells and some are aggregated (yellow, arrow). In the POAG cells (B) all autophagosomes are centered around the nuclei in a limited arc. Bar = 25um. C and D) Immunostaining for LC3 in XFS-TFs (C) and POAG-TFs (D). Arrow denotes large autophagasome aggregates in XFS-TFs. Bar = 50um. E) Western blot for LC3 I and II and quantification of LC3II pixel density/GAPDH. F) Cyto ID fluorescence by flow cytometry in XFS and POAG cells incubated for 24 h under starvation with or without chloroquine. N = 4 for XFS-TFs and POAG-TFs.

One frequent approach to the study of autophagy dynamics is the ectopic expression of fluorescently tagged LC3 [38, 39]. In particular, the advanced EGFP-mCherry tandem variant exploits the fact that autolysosomes preserve the low pH of the contributing lysosome and that at these low values only the mCherry protein remains fluorescent (GFP is quenched). Hence, the tandem construct in addition to reporting the localization of LC3-II in live cells reports the present of transient autolysosomes as red only spots. The unanchored, fully delocalized LC3-I is not visible. Fig 6A and 6B depict the fluorescence patterns of the tandem transduced XFS and POAG cells. Paralleling the distribution of endosomes and lysosomes described above, in the starvation induced XFS-TFs, autophagosomes are present both throughout the cytosol and the cell center whereas in the POAG cells there were exclusively packed around the nucleus, mostly on one side. Red puncta reporting autolysosomes are were present in both cell types and, remarkably, in the XFS they were seen far from the cell center, suggesting that the autophagosomes were fusing with lysosomes at these distant locations. Additionally, a salient feature revealed by the comparison was the accumulation of LC3-II in amorphous autophagasome globules in the XFS (yellow), which were not apparent in the POAG cells. This same feature was observed when these cells were immunostained for LC3. A substantial fraction of the LC3 immunostain in the XFS occurred in large globular structures fully absent in the POAG cells (Fig 6C and 6D). Consistent with these large intracellular LC3 accumulations, Western blots demonstrate that XFS-TFs accumulate much higher quantities of LC3 at steady-state than POAG cells (Fig 6E). In particular LC3II, the isoform associated with autophagosome content, which, due to a number of considerations is deemed a more reliable base for intra-cellular comparisons [40] was 3.91 +/- 0.50 times higher in the XFS cells than in the POAG cells, p = 0.0001.

To obtain an autophagy rate comparison (changes in autophagic flux) between the two cell types, we utilized Cyto ID, a fluorescent cationic amphiphilic tracer dye that rapidly partitions in a slowly reversible manner into intracellular organelle membranes to obtain an independent quantitative measurement of the rate of autophagosome clearance. Due to titratable functional moieties it cannot accumulate in the acidic lysosomes an autolysosomes. Properly selected dye concentration is used so that the rate of dye uptake in cellular organelles and the dye loss due to fusion of these organelles with acidic lysosomes are within the same range. The loss of signal is abolished when lysosomal acidity is reduced by introduction of chloroquine, leading to a reading of the total accumulation of dye in the cell. Thus, the fluorescence intensity difference between chloroquine-treated (total) and untreated samples is termed autophagic flux [41].

Untreated and chloroquine pretreated cells were stained with Cyto-ID dye as described in Methods. Flow cytometry measurement of the cells showed that while in the XFS cells the total arrest of autophagic flux by chloroquine caused a 30.1% (p = 0.19) increase in accumulated dye, in the POAG cells, chloroquine caused a 65.1% (p = 0.0003) increase in Cyto-ID fluorescence, which was highly significant (Fig 6F). Thus, autophagic flux in the XFS-TFs was 53% lower (p = 0.01) than that in POAG. (It is noteworthy that in these experimental series we attempted to increase the rate of autophagy by combining serum-withdrawal with the addition of rapamycin. The addition of this autophagy activator had no effect on the ± chloroquine difference, suggesting that the serum-removal maneuvers were sufficient to achieve maximal rates of autophagic flux, data not shown).

Together our data demonstrating in XFS-TFs a) buildup of LC3 aggregates in the live cells transduced with the LC3 tandem reporter and in the LC3 immunostaining of fixed cells; b) an accumulation of of LC3II by Western blot and c) reduced autophagic flux by Cyto ID quantified by flow cytometry, suggest that autophagasomes have a reduced rate of clearance from the cell.

### XFS-TFs have a high content of cells with low MMPT

A critical role of autophagy is mitophagy, the clearance of aging or otherwise damaged, mitochondria. Diminished or defective autophagy may lead to reduced clearance of decaying mitochondria with all its implications for cell health [42]. Mitochondrial decay implies diminished or complete loss of the inner mitochondrial membrane potential (MMPT) that drives ATP synthesis. One of the most effective dyes to measure this parameter is JC1, an inner mitochondrial membrane potential-sensitive dye that exhibits a concentration dependent bathochromic emission shift. Since high membrane potential enhances JC1 accumulation it leads to its aggregation, causing an increase in the 585 nm (orange-red) emission at the expense of the 530nm (green) emission that dominates the spectrum in diluted solution [43]. Thus, the MMPT characteristics of populations of TFs in either complete medium or after a starvation challenge were determined by flow cytometry as described in Methods.

**Fig 7:**
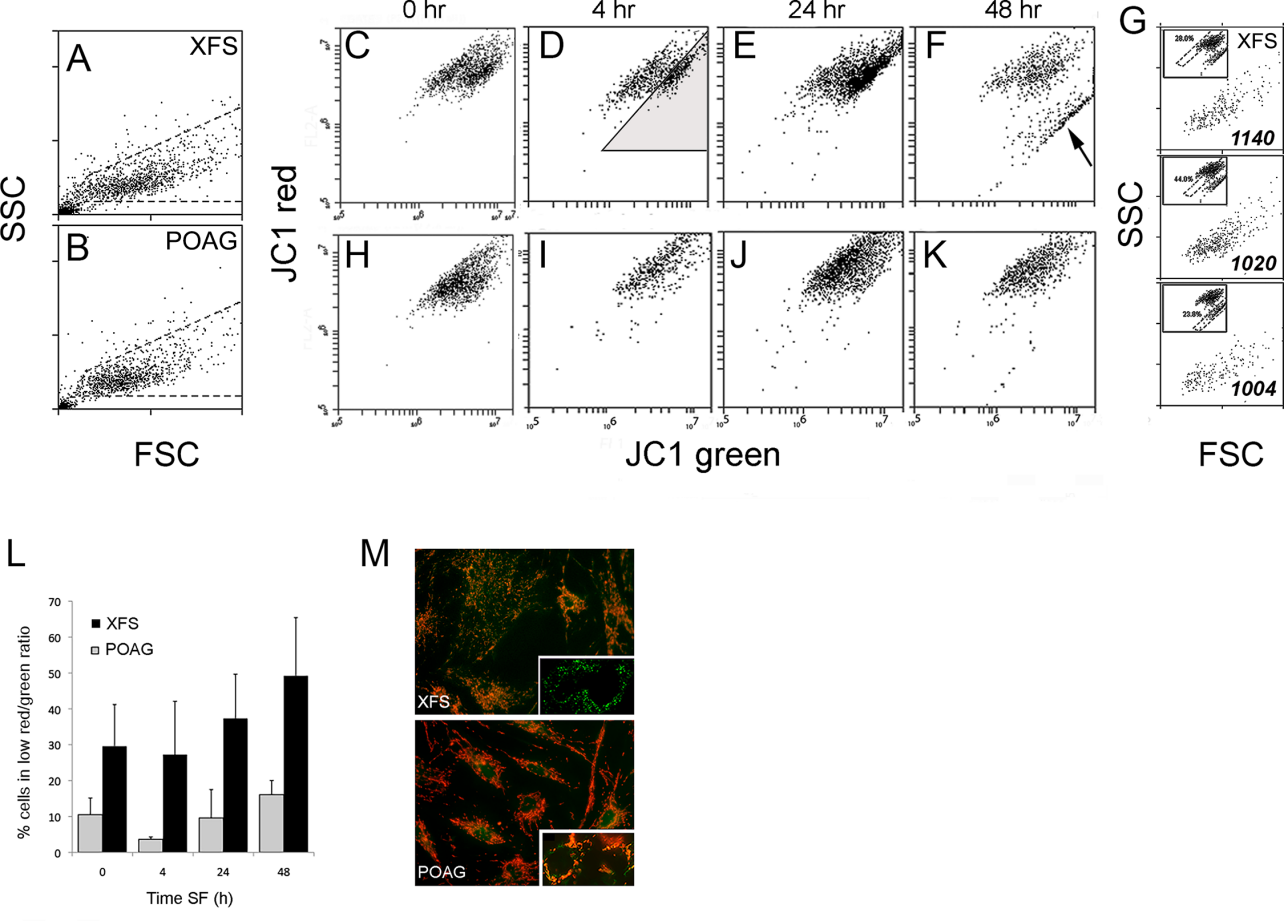
MMPT in XFS and POAG-TFs. A-B) Representative light scatter plots for, respectively, XFS and POAG cells, after 48 h culture in SSFM; the measurements of JC1 emissions were made with the cells incorporated within the dashed line gates. C-F) Orange/green emission bivariate emission plots of JC1 stained XFS-TFs in complete medium (t = 0) or 4 hr, 24 hr, or 48 hr after cell re-seeding in SSFM (starvation) medium. G) Light scatter plots from three separate distinguishable zones in the XFS cells maintained in SSFM for 48 h (sample F; each zone is defined by the gate in the respective JC1 insert). Note that both, the visual distribution in the SSC/FCS plot and the relative cell granularity (SSC arbitrary units in the right bottom corner of the SSC/FSC plots) are identical for all three cohorts. H-K) Equivalent images for POAG cells. L) Mean ± SDs of percent of XFS and POAG cells included within the low orange/green bivariate region. The region is shown in frame D as a shaded triangle. In all four culture times the percentile difference between the XFS 4 specimen set and the POAG four specimen set was highly significant (p < 0.02). M) Micrograph of JC1 stained XFS and POAG cells. Inserts show comparable pictures taken at high spatial resolution (63x objective, na = 1.4) of a live cells exhibiting fully depolarized mitochondria in the XFS population and typical cells in the POAG set.

In these experiments we increased the culture time in starvation medium to 2 days. Because of the stress introduced by the removal of serum we paid close attention to the light scattering properties of each individual sample along the culture time. No measurable increases in very low % of SSC^high^ cells (Fig 7A and 7B) were observed. Whether in complete medium or after a short 4 h exposure to starvation condition the XFS-TF populations displayed two cohorts centered around different orange/green emission ratios (Fig 7C–7F). Given that each cell contains hundreds of mitochondria, the lower ratio cohort, (the dark triangle, Fig 4D) incorporates only cells that are overtly outside the main ratio cohort. This represents cells in which either a sizable fraction of mitochondria is highly depolarized or cells in which all mitochondria possess a lower MMPT. After two days in SSFM medium, some of the XFS-TFs acquired a very low emission ratio (Fig 7E, arrow). Addition of 10 μM FCCP for 20 min to abolish all MMPT brought the whole cell population to this lowest, single ratio level suggesting that these are highly depolarized, yet alive (PI-negative) cells (data not shown). This dramatic mitochondrial depolarization after 48 h suggested that these cells, while not yet dead, maybe be undergoing apoptosis. Yet, comparison of SSC levels between the three distinguishable JC1 subpopulations in the XFS 48 h sample failed to indicate any increase in SSC (Fig 7G). Additionally, annexin V^high^ cells never exceeded 2% of the populations whether in the complete medium or after culture for 48 h in SSFM (n = 6; data not shown).

In contrast to the XFS cells, most POAG cells, displayed a single cohort whose orange/green emission ratio was equal or very similar to the higher XFS cohort at all times (Fig 7H–7K). Only a small proportion of POAG cells fell within the definition of the lower ratio XFS cohort and very low ratio cells were never observed. Fig 7L displays the mean ± SD for the % of cells falling within the low ratio zone delimited by the Fig 7D triangle. The following observations can be made. Firstly, this percent was at least three times higher both in complete medium (Fig 7L, t = 0) and in SSFM at various times. Secondly, the percent of cells with reduced MMPT increased gradually once the cells were exposed to starvation in both XFS and POAG cells but the difference in percentiles remained large and statistically significant in all cases (p< 0.02). It should be pointed out that selecting a more inclusive zone (i.e., closer to the main cohort) changed slightly the absolute numbers but not their relationship of the very high statistical significance.

To provide a visual correlation to the flow cytometry study of suspended cells, JC1- stained live cells in culture were examined in parallel in the inverted epifluorescent microscope after 24 hrs of starvation. XFS-TFs contained a proportion of cells with mitochondria displaying low red emission green emission (Fig 7M, top) and even some cells lacking all red mitochondria (insert). This pattern has also been observed in other human cells after prolonged toxic exposure [44]. POAG -TFs displayed the classical image of normal cells with the majority of their mitochondria stained red indicating a high MMPT (Fig 7M, bottom).

## Discussion

Here we report for the first time that dysfunction in endo- and lysosomal positioning, autophagy, and mitochondrial health may be components of XFS pathology. Our initial findings demonstrate that XFS-TFs are larger than controls, and do not properly organize in 3D culture (Figs 1 and 2). Furthermore, in contrast with the relocation responses of lysosomes seen in POAG-TFs and no glaucoma-TFs from young donors, XFS-TFs lysosomes failed to relocate to the perinuclear area in response to removal of serum (Figs 3 and 4). The similarity between TFs from old POAG and TFs from young donors not suffering from glaucoma, when compared with the differences between them and the XFS cohort support our finding that although aging generally reduces the cellular capacity for autophagy [45], age is not the sole determinant generating these large differences observed between XFS and POAG cells. Supporting our data on dysfunctional lysosomal localization after serum-withdrawal, we also demonstrate that in XFS-TFs early, recycling, and late endosomes have strikingly altered distributions (Fig 5) and autophagic flux is reduced (Fig 6). Additionally, the deficiencies observed in mitochondria (Fig 7) reveal impaired mitophagy. This latter impairment causes accumulation of damaged mitochondria that may have serious implications for the cell ability to manage oxidative insult and/or bioenergetic management [18]. Together our data support the hypothesis that age-induced autophagic/mitochondrial dysfunction combined with underlying genetic causes of XFS disease may induce XFS pathology.

A key marker of aging cells is reduced polymerization of microtubules that control the positioning of lysosomes and mitochondria in the cell leading to ineffective cellular degradation and in turn, oxidative stress [28, 46]. A defect in vesicle positioning, leading to reduced degradation of cellular waste, has been identified in neuronal diseases such as Alzheimer’s, Huntington’s and Parkinson’s [14, 30, 47–49]. These phenomena appear to be linked to or enhanced by age-related changes in microtubule polymerization. There is a large body of evidence suggesting that in Alzheimer’s for instance, Aβ accumulation in the extracellular space results at least in part from a decrease in fusion of endocytic vesicles that contain Aβ peptides with lysosomes because of disruptions in microtubule dynamics, resulting in enhanced secretion of Aβ peptides and thus, plaque formation [14]. Consistent with these observations, compared to POAG and young healthy controls microtubule organization centers in XFS-TFs are often not apparent (Fig 3). Since substantial amount of ATP are needed for microtubule dynamics, vesicle repositioning and autophagic flux, the compromised mitochondrial function in XFS cells may contribute to disturbances in microtubule dynamics. Another parallel with Alzheimer’s is a recent study demonstrating that increased activation of the early endosome marker, rab5 causes enlargement of early endosomes and disruption of retrograde neuronal trafficking [50]. Interestingly, rab5-positive endosomes in XFS-TFs are very large and mislocalized suggesting that this pathway may also be of interest in XFS pathology.

Another potentially important marker of dysfunction is the accumulation of Fibulin-5 in XFS-TFs after long-term culture self- assembling conditions (Fig 2). It is intriguing that not LOXL1 but rather Fibulin 5, its main associate protein in the synthesis of the elastin fibers that undergo intracellular aggregation and seemingly abnormal over-accumulation. Further characterization of the XFS fibroblasts maintained in long term culture will clarify whether the site of accumulation contains only Fibulin 5 or other proteins identified in the intracellular XFM aggregates. The question as to whether the aggregates form within the cell or represent endocytosed extracellular debris that challenges the cell degrading machinery is another substantial question that will be examined. Such studies may help to find the cellular and molecular origins of the XFS extracellular aggregates.

Two external observations support the conclusions detailed above regarding the link of XFS to defects in autophagy and/or lysosomal disease. Firstly, in mice, the absence of the lysosomal trafficking regulator gene Lyst, a protein required for normal lysosomal function, elicits multiple features of XFS including, saw tooth morphology of the iris pigment epithelium, XFM-like aggregates, pigment dispersion and high levels of oxidative stress [51, 52]. Secondly, XFS is strongly age related, a near indispensable factor in the development of autophagic deficiencies. Research in several neuronal or retinal diseases has documented lysosomal phenotypes. In AMD reduced lysosomal acidity results in a build-up of internal cellular waste that leads to cellular bloating. Accordingly, lowering the pH of lysosomes, a strategy that has been suggested to slow or prevent the progression of AMD [53, 54], may also provide a means for the treatment of XFS.

In summary, our data suggest that dysfunction in the degradative processes inside the cell compounded by an ensuing build- up of non-functioning mitochondria and interferences with microtubule dynamics may contribute to XFS pathology. These data align with data from other systems in which autophagy impairment in lysosomal disease involves cross talk with increased oxidative stress and accumulation of effete mitochondria [16, 18]. This correlation with other retinal and neurodegenerative diseases may open the door for novel therapies, some of which may already be under development for the treatment of other age-related diseases.
